# ER expression associates with poor prognosis in male lung squamous carcinoma after radical resection

**DOI:** 10.1186/s12885-021-08777-6

**Published:** 2021-09-21

**Authors:** Xue Yang, Xiangfeng Jin, Rongjian Xu, Zhuang Yu, Ning An

**Affiliations:** 1 Department of Medical Oncology, The Affiliated Hospital of Qingdao University, Qingdao, 266003 Shandong China; 2 Department of Thoracic Surgery, The Affiliated Hospital of Qingdao University, Qingdao, 266003 Shandong China; 3 Department of Radiation Oncology, The Affiliated Hospital of Qingdao University, Qingdao, 266003 Shandong China

**Keywords:** Estrogen receptors, Male patients, Lung squamous carcinoma, Prognostic biomarker, Therapeutic targets

## Abstract

**Background:**

Clinical options for lung squamous carcinoma (LUSC) are still quite limited. Carcinogenesis is an exceedingly complicated process involving multi-level dysregulations. Therefore, only looking into one layer of genomic dysregulation is far from sufficient.

**Methods:**

We identified differentially expressed genes with consistent upstream genetic or epigenetic dysregulations in LUSC. Random walk was adopted to identify genes significantly affected by upstream abnormalities. Expression differentiation and survival analysis were conducted for these significant genes, respectively. Prognostic power of selected gene was also tested in 102 male LUSC samples through immunohistochemistry assay.

**Results:**

Twelve genes were successfully retrieved from biological network, including ERα (ESRS1), EGFR, AR, ATXN1, MAPK3, PRKACA, PRKCA, SMAD4, TP53, TRAF2, UBQLN4 and YWHAG, which were closely related to sex hormone signaling pathway. Survival analysis in public datasets indicated ERα was significantly associated with a poor overall survival (OS) in male LUSC. The result of our immunohistochemistry assay also demonstrated this correlation using R0 resected tumors (*n* = 102, HR: 2.152, 95% CI: 1.089–4.255, *p* = 0.024). Although disease-free survival (DFS) difference was non-significant (*n* = 102, *p* = 0.12), the tendency of distinction was straight-forward. Cox analysis indicated ERα was the only independent prognostic factor for male patients’ OS after R0 resection (HR = 2.152, *p* = 0.037).

**Conclusion:**

ERα was significantly related to a poor prognosis in LUSC, especially for male patients after radical surgery, confirmed by our immunohistochemistry data.

**Supplementary Information:**

The online version contains supplementary material available at 10.1186/s12885-021-08777-6.

## Background

Lung squamous carcinoma (LUSC) accounts for approximately 20 to 30% of all lung cancers and is associated with poor prognosis compared with non-squamous non-small-cell lung cancer (NSCLC) [1–3]. Up to date, unlike lung adenocarcinoma, targeted therapeutic choices for LUSC are still relatively scarce. Before the era of immunotherapy, platinum-based chemotherapy seemed to be the most proper option of first-line treatment for advanced stage LUSC patients [4, 5]. It has been widely reported that immune check point inhibitors, like pembrolizumab, atezolizumab, and nivolumab, can substantially improve LUSC patients’ prognosis [6–8]. However, the subsequent strategies after failure with immunotherapy were enormously limited. Therefore, prognostic biomarkers and candidate therapeutic targets are greatly needed for this deadly disease.

The functions of estrogen and estrogen receptors (ERs) have been intensively studied in reproductive organs, under most circumstances, in females. The importance of ER signaling pathway has also been extensively investigated in various pathologic functions during carcinogenesis, especially in breast cancer. There are two types of classic ERs. i.e. ER alpha (ERα, also known as ESR1), located on Chromosome 6, first cloned in 1986; ER beta (ERβ, also known as ESR2), located on Chromosome 14, first cloned in 1996 [9]. Estrogen is also believed to play an important role in lung carcinogenesis [10, 11]. Some researchers have already investigated the correlation between ER and lung cancer based on immunohistochemistry stain, but the results of these researches seem quite confusing. Some studies showed that ERα in lung cancer was mainly observed in the cytoplasm and associated with a poor prognosis. Some found that the nuclear ERβ predicted a better prognosis, and the expression level of cytoplasm was associated with a poor prognosis [12–15]. Nonetheless, opposing results have also been widely reported in various studies [16–19]. Moreover, the majority of these researches focused on female patients [20–22], probably subjected to the stereotypical thinking that estrogen plays a more important role in women. Additionally, according to literature review, ER-related investigations in lung cancer were majorly concentrating upon adenocarcinoma, seldom paying enough attention to LUSC, except for some subgroup analyses among NSCLC, let alone male LUSC patients. Therefore, the relationship between ER and the prognosis of male LUSC is greatly needed in order to provide a potential therapeutic target for these patients, since the majority of the LUSC patients are male.

It is putatively accepted that the process of carcinogenesis is due to multi-level abnormalities, including genetic alterations [majorly composed of somatic mutation and copy number variation (CNV)] [23, 24], aberrant methylomics [25, 26], and dysregulated transcriptomics [27, 28]. CNV, promoter methylation and somatic mutation could all influence gene activation or suppression, respectively, thereby leading to cancer transformation. CNVs may alter transcriptional dosage by influencing the number of copies of a gene located within a particular genomic region [29–32], explaining under most circumstances, CNV and corresponding gene expression are positively correlated in lung cancer [33–35]. Promoter hypomethylation might lead to gene activation, and promoter hypermethylation might cause gene suppression in most cases [36]. For instance, *MGMT* silencing, caused by its promotor methylation, can make glioma patients greatly benefit from temozolomide [37]. Somatic mutation could also result in activation or suppression of downstream signaling pathways [38]. In thyroid cancer, *BRAF* V600E mutation could activate *MAPK* pathway, thus influencing the massive dysregulation of molecular interactions [39]. Therefore, the multi-dimensional genomic abnormalities should be taken in to consideration aggregately, rather than only looking into the gene expression differentiation, in order to shed light upon the intricate bio-mechanisms of cancer transformation and progression. The integrative analysis of cancer genomics, methylomics and transcriptomics is a very wise choice to comprehensively dissect cancer etiology and provide potential clinical guidance of LUSC.

## Methods

### Data collection and organization

The multi-level datasets of LUSC were directly downloaded from the Bioconductor package “RTCGA.rnaseq” and “TCGAbiolinks” at January 10th, 2020. Three levels of paired data (cancer and normal adjacent tissues) were retrieved from all patients, including 51 paired RNA sequencing data [raw counts and RNASeq by Expectation Maximization (RSEM) normalized read counts], 237 paired DNA CNV data (generated from Affymetrix SNP 6.0 platform, and segmented through circular binary segmentation method [40]), 40 paired DNA methylation data [generated from Illumina HumanMethylation450 platform, and the methylation level of each CpG site was calculated as the ratio (β value) of signal of methylated probes relative to the sum of all probes, ranging from 0 (absolutely unmethylated) to 1 (fully methylated)], and somatic mutation data of 548 patients (with mutational information of 17,225 genes). The clinical information of these The Cancer Genome Atlas (TCGA) LUSC patients was also directly downloaded from this Bioconductor package.

The mRNA expression profiles of LUSC, including GSE73403, GSE37745, GSE41271 and GSE50081, were downloaded from Gene Expression Omnibus (GEO) database, and the final normalized datasets were used in following analysis. All clinical information was extracted from the original publications.

### Identification of significant dysregulated genes

Differentially expressed genes (DEGs) were identified using paired *t* test with RSEM normalized read counts (*FDR* < 0.01). Bioconductor package “CNTools” was used to process segmentation CNV data, and transform the segmentation data into a gene-level matrix, according to each genomic location of 27,270 genes. Dysregulated genes with significant amplification and deletion were also identified using paired *t* statistic test (*FDR* < 1*10^− 5^). Promoter region was defined as the genomic length between 1000 bps upstream transcription start site (TSS) and 300 bps downstream. In methylation analysis, the β value of the probe mapped to the certain CpG site within promoter region of a given gene was used to determine the methylation level of this probe. If multiple probes were projected onto the promoter region of a given gene, the mean value was used to define the methylation value of this gene. The methylation level of 20,110 genes were obtained in this manner, and significant hypo- or hyper-methylated genes were identified with paired *t* statistic test (*FDR* < 0.01).

Therefore, three groups of candidate genes of interest were identified for further analysis by virtue of different dysregulation patterns: (i) genes with both differential expression and consistent CNV (i.e. over-expressed genes with amplification, and under-expressed ones with deletion); (ii) genes with both differential expression and altered promoter methylation (i.e. over-expressed genes with promoter hypomethylation, and under-expressed ones with significant promoter hypermethylation); and (iii) genes with both differential expression and somatic mutation (mutation rate ≥ 5%).

### Identification of significant genes using random walk algorithm in biological network

We downloaded protein–protein interaction network was from Human Protein Reference Database (HPRD) and Kyoto Encyclopedia of Genes and Genomes (KEGG) database. Gene regulatory network was therefore constructed by merging HPRD and KEGG network together, including 10,479 nodes and 60,689 edges after eliminating self-loops and duplicated edges.

In this study, random walk algorithm was utilized to identify genes significantly affected by genomic and epigenetic alterations, taking advantage of aforementioned merged biological network [41]. The similar method has been successfully conducted in our previous study [42]. Genes of interest were referred to as information source (i.e., source nodes) and the rest as the information target (i.e., target nodes). The information flow originates from source nodes iteratively and randomly transmits to their neighbors with a probability proportional to their topological features. At each iteration, information flow can go back to source nodes and target nodes as well with the same probability. The final steady-state probability assigned to each gene stands for the integrated influence coming from source nodes while considering the network topology. Formally, the random walk with restart can be illustrated as followings:

$$ {p}^{t+1}=\left(1-r\right){Wp}^t+{rp}^0 $$

Where *W* stands for the column-normalized adjacency matrix of connected network, and *p*^*t*^ is a vector of probability the genes in the network holds at step *t* in the iteration process. Source nodes were weighted with initial probability vector *p*^*0*^ (the sum of its elements was normalized as 1), and *r* represents restart probability (*r* = 0.7 in the present study). All the genes in the network were eventually scored according to the values in the steady-state probability vector *p*^∞^. This was obtained at query time by repeating the iteration process until the difference between *p*^*t*^ and *p*^*t + 1*^ (measured by the L1 norm) was lower than 10^− 10^. To obtain genes with significantly high steady-state probability, 10,000 permutations of node labels (with network topology remained the same) were performed to simulate the null distribution of final probability. The *p* value was termed as the probability of random values that were less than the calculated final probability. Genes with *p* < 0.02 were designated as the genes significantly afflicted by these genetic or epigenetic abnormalities.

### Immunohistochemistry to identify the expression level of ER and androgen receptor (AR) in male LUSC tumors

One hundred and two formalin-fixed paraffin-embedded (FFPE) primary LUSC samples with OS and DFS information were collected after radical surgery in Department of Thoracic Surgery from January 2014 to December 2016. All the patients were male, ranging from IA to IIIA based on AJCC 7th staging system. The clinicopathological factors were carefully documented, including age, pathological tumor size, regional lymph node metastasis, AJCC stage, grade, whether adjuvant chemotherapy or radiotherapy were conducted. None of these patients received any kinds of neoadjuvant therapy. Anatomic pulmonary and mediastinal lymph node resection were all adopted in these 102 patients, and both squamous phenotype and R0 resection were confirmed by experienced pathologists.

Slides of the various male LUSC tumors were stained with anti-ERα (working reagent, Zhongshan Bio-chemistry, China), anti-AR (working reagent, Zhongshan Bio-chemistry, China). After incubation with anti-mouse secondary antibody (working reagent, Zhongshan Bio-chemistry, China), staining was developed using PV-6000 detecting system, and slides were counterstained with hematoxylin. Digital images of these slides were acquired using the Olympus BX37 microscope. Individual slides were scored by two independent, blinded and experienced pathologists.

### Statistical analysis

All the data analyses were conducted using R programming (Version 3.3.1) and Bioconductor packages. Gene Ontology (GO) analysis was conducted via the DAVID bioinformatics tool (http://david.abcc.ncifcrf.gov/). The information of gene-to-gene interactions was retrieved from Bioconductor package “KEGGgraph” (Version 1.50.0). Network visualization was conducted using Cytoscape software (Version 3.4.0). In survival analysis of mRNA expression data, patients were divided into two groups based on median of expression values, and Kaplan–Meier analysis and log-rank test were performed to evaluate the prognostic difference between the two assigned groups.

## Results

**Fig. 1:**
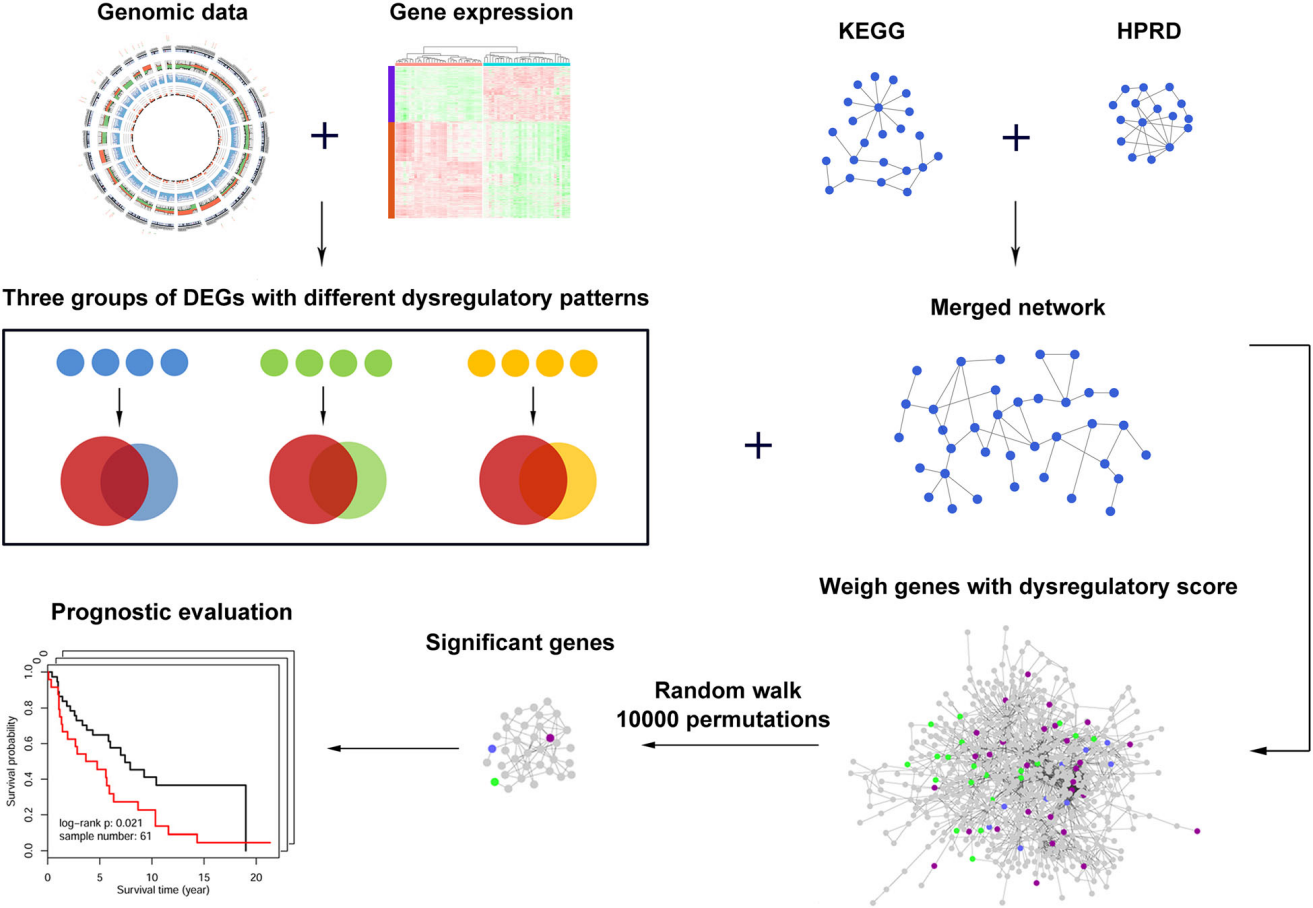
Schematic of methodology applied in this study

A schematic for the study is depicted in Fig. 1.

### Collection of genes with differential expression, CNV, promoter methylation and somatic mutation with TCGA LUSC data

**Fig. 2:**
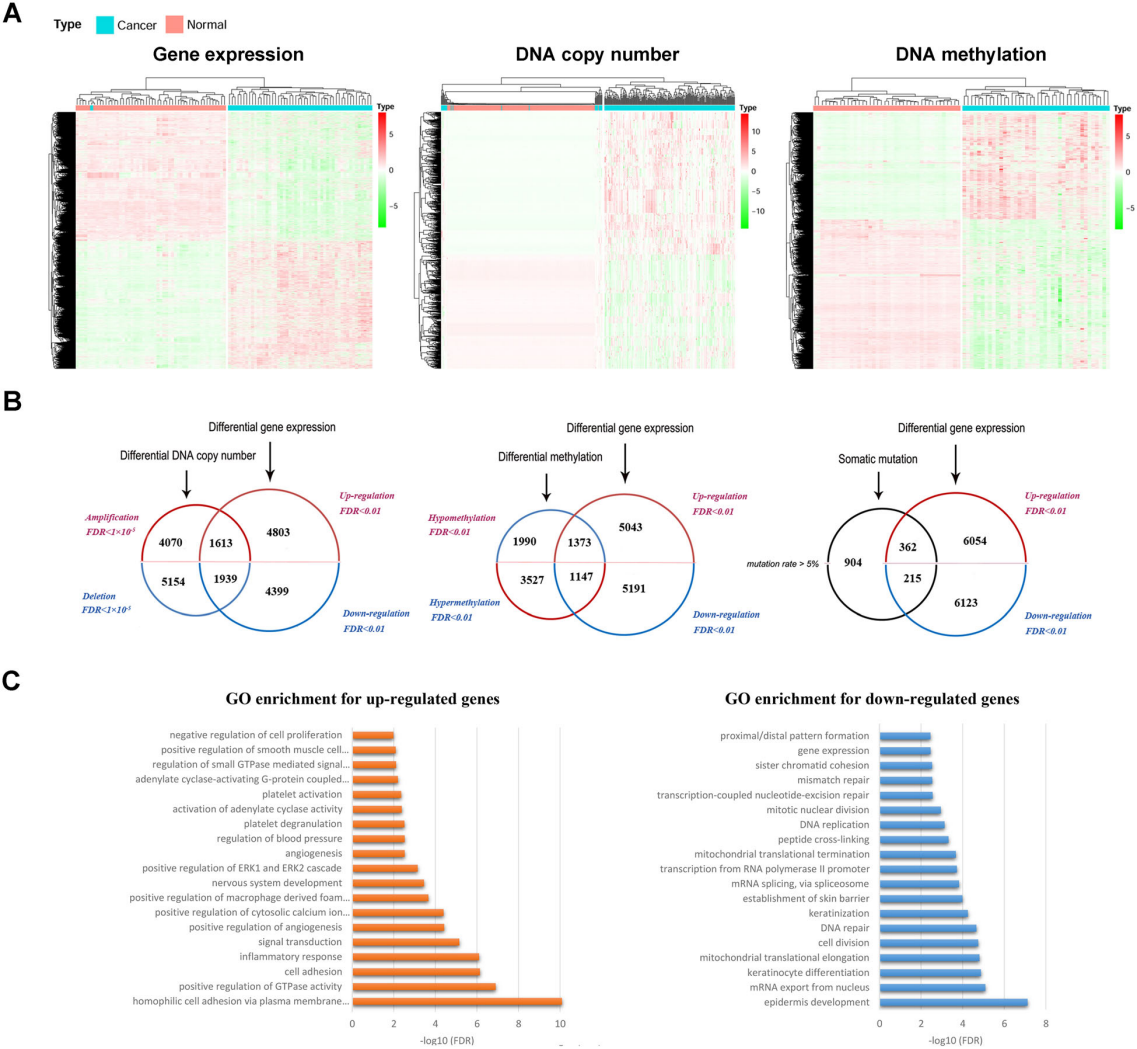
Identification of three gene groups with multi-omics data. **A** Heat maps of DEGs, genes with CNV, and genes with aberrant promoter methylation, respectively. **B** Venn diagram illustrating three groups of candidate genes with differential expression and corresponding upstream altered molecular levels. **C** GO enrichment analysis of upregulated and downregulated DEGs with at least one types of consistent genetic or epigenetic dysregulations

Paired samples were used to eliminate individual difference. DEGs were identified with paired *t* test, composed of 6416 up-regulated genes and 6338 down-regulated genes (Fig. 2A). Additionally, 5683 genes were identified as significantly amplified and 7093 genes were found deleted based on paired CNV data (Fig. 2A). Furthermore, 4674 genes with promoter hypermethylation and 3363 genes with hypomethylation were also identified as significant according to paired methylation profiles (Fig. 2A). Moreover, 1481 genes with mutation rate ≥ 5% were deemed as mutated genes with relatively high frequency.

### Identification of candidate gene groups with different dysregulation patterns

Three different groups of DEGs with aberrant upstream dysregulations (Fig. 2B) were categorized as follows: (i) 1613 genes with over-expression and copy number gain, and 1939 genes with under-expression and copy number loss (altogether 3552 genes, termed as Group A); (ii) 1373 genes with over-expression and promoter hypomethylation, and 1147 genes with under-expression and promoter hypermethylation (altogether 2520 genes, termed as Group B); (iii) 577 DEGs (termed as Group C) with somatic mutation in more than 5% patients (362 over-expression and 215 under-expression). Genetic and epigenetic dysregulation of DEGs were shown in Fig. 2B. To comply with classic knowledge of gene regulation, promoter methylation was assumed to exert trans-regulation, while DNA copy number did cis-regulation effect upon transcriptomics.

Up-regulated and down-regulated DEGs, within Group A, B or C (with at least one types of consistent upstream dysregulations), were both used to conduct GO enrichment analysis, respectively. The results indicated that up-regulated DEGs were significantly associated with cell adhesion, positive regulation of GTPase activity, inflammatory response, and positive regulation of angiogenesis (Fig. 2C). Down-regulated DEGs were significantly related to epidermis development, mRNA exportation, cell division, and DNA repair (Fig. 2C).

### Genetic alterations in LUSC

**Fig. 3:**
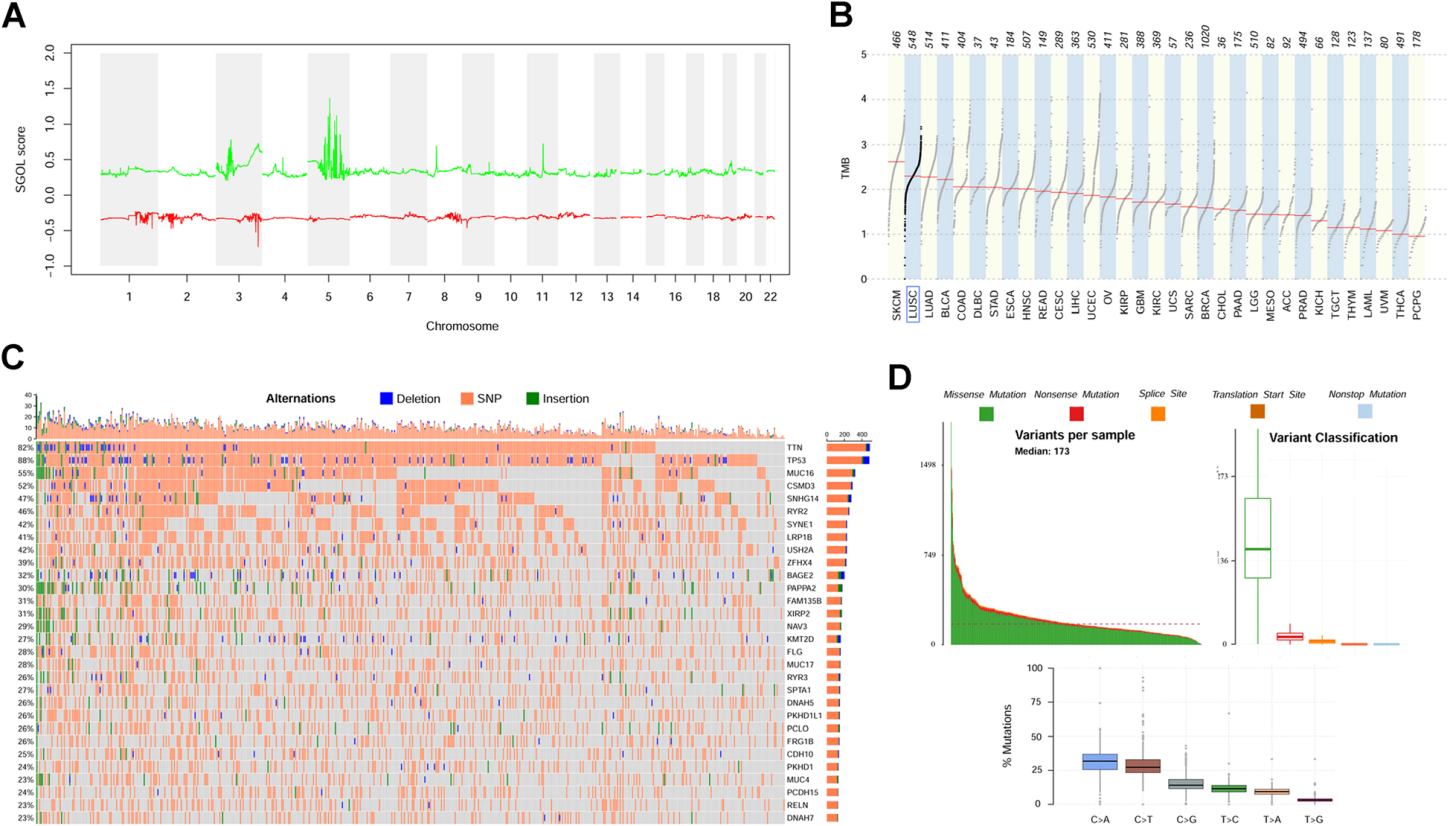
Genetic alterations in LUSC patients. **A** SGOL scores of LUSC in 22 autosomes. **B** TMB analysis of all the cancer types in TCGA datasets. **C** Oncoprint plot of top 30 genes mutated most frequently in LUSC. **D** Display of related information regarding somatic mutation

Segment Gain or Loss (SGOL) scores were calculated based on the segmentations mapping onto 22 autosomes. Since the TCGA samples we used were a mixture of male and female patients, we chose to drop the data on X and Y chromosomes to obliterate sexual difference. CNVs were observed quite frequently in Chromosome 3 and 5, which was highly consistent with previous reports [43–47] (Fig. 3A). Tumor mutational burden (TMB) analysis indicated that LUSC patients harbored the second mutation load among all the cancer types in TCGA datasets, with the log10 value of median mutations per cohort 2.30 (Fig. 3B). There were 335 genes with mutational rate > 10%, and 54 genes with mutational rate > 20% among all patients. For instance, the most frequently mutated gene was TP53, which mutated in 87.91% LUSC patients; while, the second one was TTN, with mutational rate 82.49% (Fig. 3C). The large-scale genetic alteration can be deemed as catastrophic in promoting LUSC formation and progression.

### Random walk in HPRD-KEGG merged biological network

**Fig. 4:**
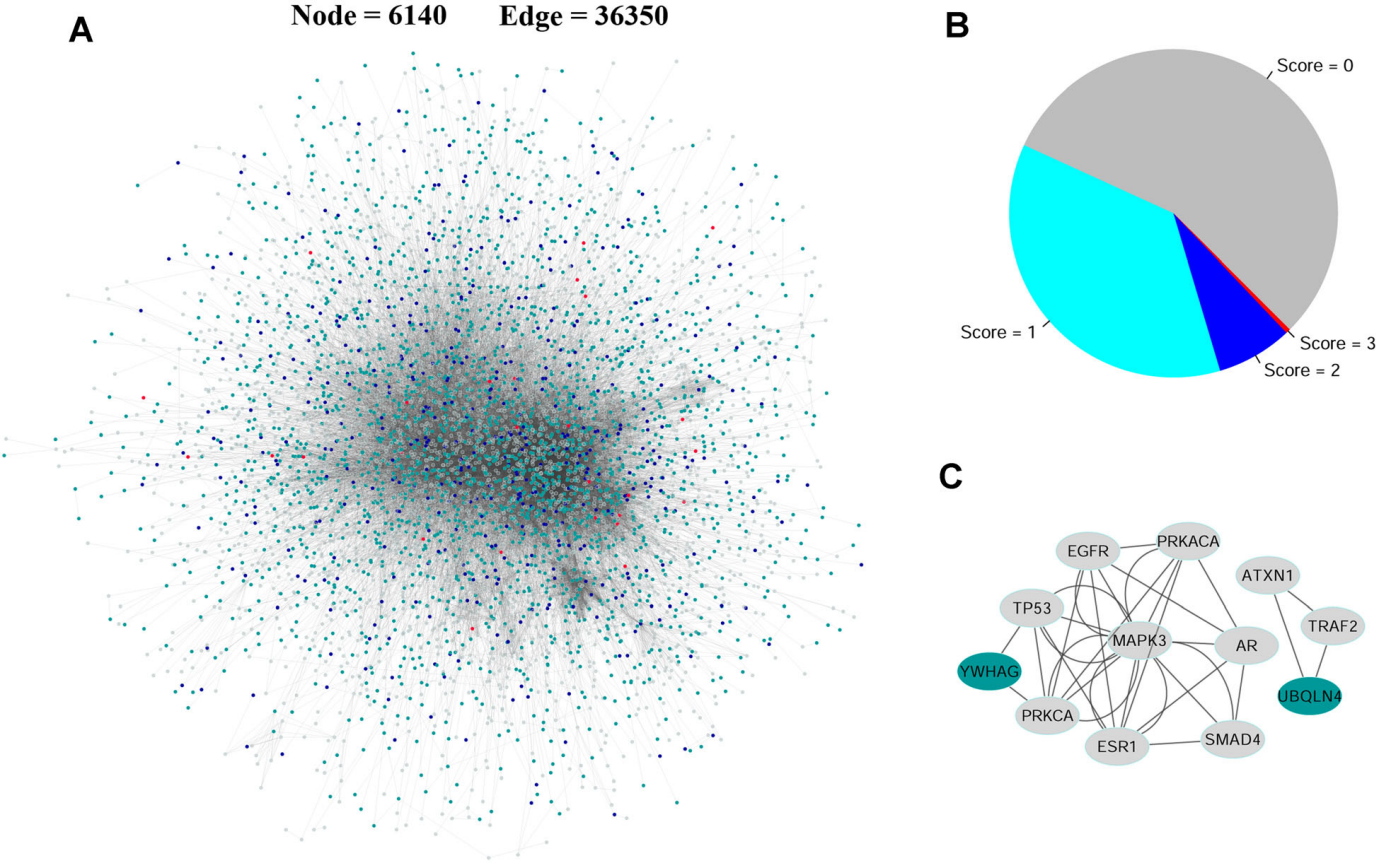
Random walk of DEGs with at least one types of upstream dysregulations. **A** The biggest connected component containing 6140 DEGs and 36,350 edges. DEGs with genetic or epigenetic dysregulations were regarded as source nodes, and the rest were target nodes. Turquoise nodes represent source genes with score 1; blue ones represent source genes with score 2; red ones represent source genes with score 3; and grey ones represent source genes with score 0. **B** Pie chart of DEGs with different score. The number of DEGs with score 1 was 2236, the ones with score 2 was 4589, and DEGs with score 3 in the network was 27. The rest 3418 DEGs (scored as 0) were target nodes. **C** The subgraph composed of 12 significant genes retrieved via random walk with restart

We first projected all the upregulated and downregulated DEGs onto HRPD-KEGG merged biological network, and then the biggest connected component (BCC), containing 6140 nodes and 36,350 edges, was established as walking graph for random walk (Fig. 4A). Genes present in the BCC belonging to Group A, B or C (2,722 genes) were used as source nodes. Genes within only one gene group (afflicted with one consistent dysregulation, i.e. dysregulated promoter methylation, CNV or somatic mutation) were scored as 1 (*n* = 2236); genes simultaneously belonging to two gene groups (afflicted with two consistent dysregulations) were scored as 2 (*n* = 459); and genes within all three gene groups were scored as 3 (*n* = 27) (Fig. 4B). The initial probability vector was obtained by normalizing the score vector so that the sum of the vector was equal to 1. Therefore, 12 significant genes were successfully retrieved in respect to steady-stage probability through 10,000 permutations (Fig. 4C). These 12 significant genes were closely related to sex hormone signaling pathway, containing both ERα (ESRS1) and AR molecules.

### Expression differentiation and survival analysis of ER and AR

**Fig. 5:**
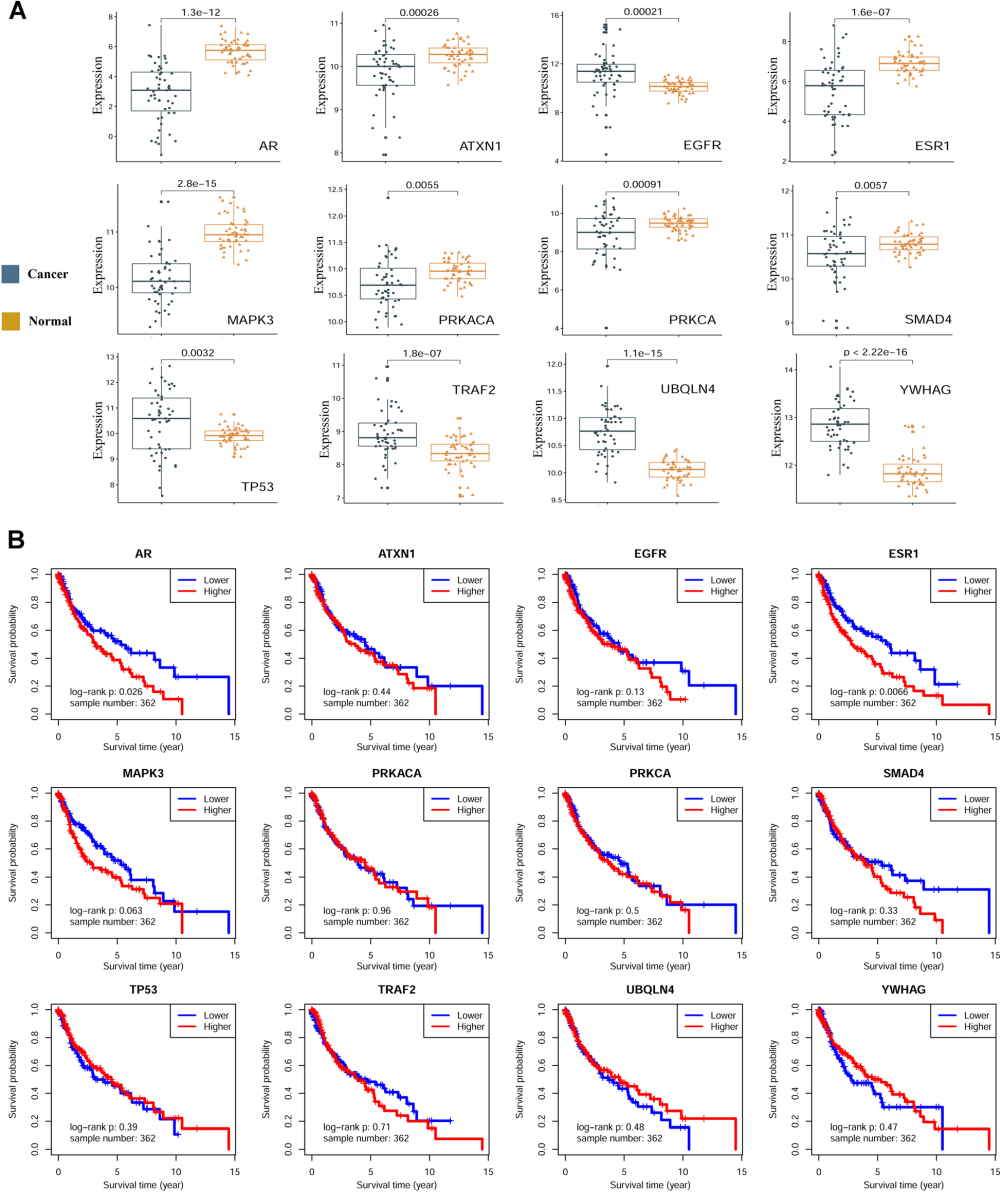
Expression differentiation and OS association of 12 significant genes. **A** Boxplot of these 12 significant genes in paired samples, and corresponding *p* values of paired t tests were also displayed. **B** OS analysis of these 12 significant genes in male patients

Paired *t* test indicated that all these 12 significant genes were significantly differentiated between caner and normal adjacent tissues (Fig. 5A), and the same tendency were accordantly observed in both male and female LUSC patients, respectively (Supplementary Figures S1-S2). We collected 490 patients with OS information, and survival analysis showed that the expression level of ERα was significantly associated with LUSC patients’ OS (*p* = 0.0025, Supplementary Figure S3). Although the survival analysis didn’t reach significance threshold, AR’s expression showed a strong relation with patients’ OS (*p* = 0.13, Supplementary Figure S3). The survival distinction seemed blurred in 128 female LUSC patients (Supplementary Figure S4), while higher expressions of ER and AR were both significantly associated with poor OS (*p* = 0.0066 for ER, *p* = 0.026 for AR, respectively) in male patients (Fig. 5B). We thus collected the disease-free survival (DFS) information of 174 male LUSC patients, who were confirmed suffering with local disease and undergoing R0 radical resection according to TCGA clinical data. Survival analysis of AR and ER showed a tendency in predicting a poor DFS in male patients, despite not reaching significance level (*p* = 0.27 for AR, *p* = 0.24 for ER, respectively, Supplementary Figure S5).

Four LUSC expression profiles in GEO database were downloaded, including GSE73403, GSE33745, GSE41271, and GSE50081, in order to show the OS association of AR and ER expression. The result indicated that ER expressions were all strongly associated with male LUSC patients’ OS (GSE73403, *n* = 65, *p* = 0.033; GSE33745, *n* = 46, *p* = 0.1; GSE41271, *n* = 48, *p* = 0.063; GSE50081, *n* = 24, *p* = 0.061; Supplementary Figure S6). However, the associations between AR and male patients’ OS, or between these two sex hormone receptors and female patients’ OS, were uncertain according to these four GEO datasets, probably because of the small sample size (Supplementary Figure S6).

### Immunohistochemistry results of ER expression in male patients

**Fig. 6:**
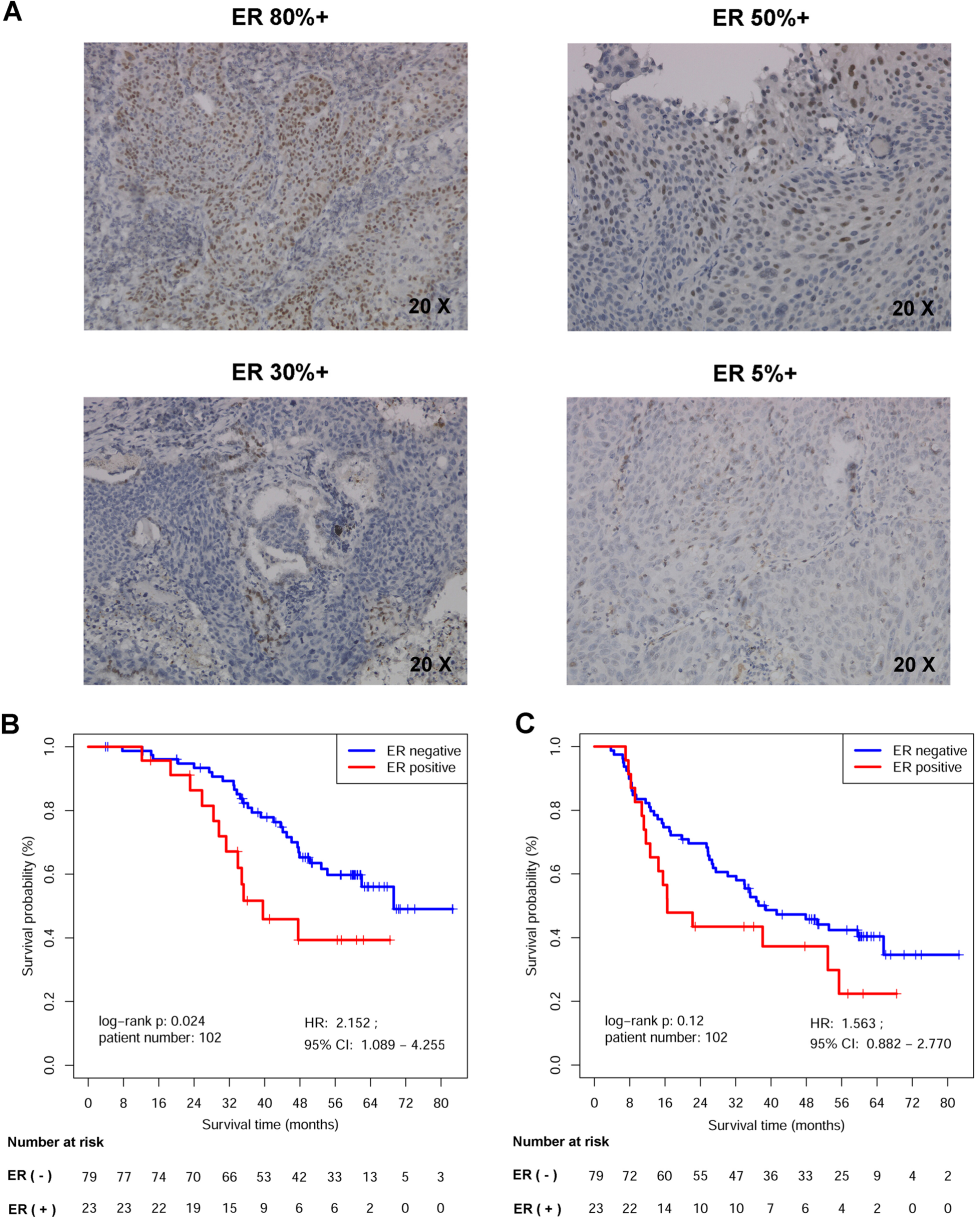
Immunohistochemistry results of ER expression in male patients. **A** ERα expression in male LUSC patients. ERα expression was mainly observed the nucleus of tumor cells, and photographs of different ER expression levels were displayed. **B** OS analysis of ERα expression in 102 male patients. **C** DFS analysis of ERα expression in 102 male patients

Out of 102 male LUSC FFPE tumors collected in our center, only 23 samples (22.55%) were regarded as ERα positive (expressed in ≥1% tumor cells). ERα expression was mainly observed the nucleus of LUSC tumor cells, which was consistent with previous researches [48, 49] (Fig. 6A). Table 1 showed the clinical characteristics of these patients [including age, pathologic tumor size (pT), regional lymph node invasion (pN), AJCC stage, pathologic grade, whether the patients underwent adjuvant chemotherapy (AdjCTX) or adjuvant radiotherapy (AdjRAD)], and the patient numbers in each factor were all balanced in ER (+) and ER (−) groups according to Chi-squared tests. Median follow-up time was 46.8 months, ranging from 3.9 to 82.7 months. Survival analysis indicated that male patients with ER positive were significantly associated with a poor OS (*p* = 0.024, Fig. 6B), and the overall HR was 2.152 (95% CI: 1.089–4.255), while the DFS difference between the two groups was not significant (*p* = 0.12, Fig. 6C). Cox analysis indicated that the ERα expression was an independent prognostic factor for male LUSC patients’ OS after radical R0 surgery (Table 2).

**Table 1:** Clinicopathological characteristics in ER positive and negative patients

Characteristics	ER expression level		***p*** value
	ER positive	ER negative	
**Age (years old)**			
≥ 65	9	19	
< 65	14	60	0.246
**pT**			
T1	8	28	
T2	8	30	
T3	4	13	
T4	3	8	0.978
**pN**			
N1	4	18	
N2	4	15	1
**AJCC stage**			
Stage I	8	27	
Stage II	8	28	
Stage III	7	24	0.998
**Pathologic grade**			
G I + II	14	51	
G III	9	28	0.938
**AdjCTX**			
Yes	15	52	
No	8	27	1
**AdjRAD**			
Yes	7	24	
No	16	55	1

^*^ Sgnificant values were in bold (*p* < 0.05). Abbreviations: AdjCTX: adjuvant chemotherapy; AdjRAD: adjuvant radiotherapy

**Table 2:** Univariate analyses of OS (Cox proportional hazards regression model) in 102 male LUSC patients

Factors	Univariate Cox regression		
	HR	95% ***CI***	***p*** value
**Age (years old)**			
< 65	Reference	–	–
≥ 65	1.735	0.918 ~ 3.279	0.100
**pT**			
T1 + T2	Reference	–	–
T3 + T4	1.360	0.704 ~ 2.627	0.369
**pN**			
N0	Reference	–	–
N+	1.217	0.653 ~ 2.268	0.539
**Stage**			
I + II	Reference	–	–
III	1.401	0.725 ~ 2.709	0.327
**Grade**			
I + II	Reference	–	–
III	1.410	0.757 ~ 2.626	0.284
**AdjCTX**			
no	Reference	–	–
yes	1.637	0.819 ~ 3.272	0.149
**AdjRAD**			
no	Reference	–	–
yes	1.401	0.725 ~ 2.709	0.327
**ER**			
ER-	Reference	–	**–**
ER+	2.152	1.089 ~ 4.256	**0.037**

^*^ Sgnificant values were in bold (*p* < 0.05). Abbreviations: *HR* hazard ratio, *CI* confidence interval, *AdjCTX* adjuvant chemotherapy, *AdjRAD* adjuvant radiotherapy

## Discussion

High-throughput and multi-dimensional genomic data usher us into a new era with overwhelming amount of information, and thus the complicated molecular mechanisms of carcinogenesis can be perceived and analyzed in a more integrative perspective. In this study, multi-level genetic or epigenetic data of LUSC, including CNV, somatic mutation, DNA promoter methylation and gene expression, were systemically utilized to discover novel and pivotal molecules and signaling pathways in a more comprehensive manner. First of all, paired samples were collected to identify significant genes with expression differentiation, CNV, promoter methylation and somatic mutation, respectively, and thereby three groups of DEGs were identified with different upstream deregulatory patterns complying with putatively accepted regulatory principles. Additionally, we adopted a simple and effective computational strategy to randomly walk DEGs with significant genetic or epigenetic alterations in HPRD and KEGG merged biological network. Random walk with restart was used to identify significant genes with the most influence from upstream abnormalities in priori knowledge-based network, of which the performance was proven to be much more superior to other methods, such as neighborhood approaches. We adopted with this strategy because it can subtly combine the multi-omics information with biological regulatory network, tracing down the information flow which meant to accumulate in significant genes. As we know, the dysregulated molecular interactions were the joint effort made by multi-level genomic malfunctions. It should be much better to take all the multi-omics data available into consideration, rather than analyze unidimensional data separately each time.

GO enrichment analysis indicated that the up-regulated DEGs with consistent upstream dysregulations were significantly associated with cell adhesion, inflammatory response, and angiogenesis, while down-regulated DEGs were mainly related to cell division and DNA repair. In some occasion, cancer cells need to adhere avidly to basement membrane matrix through upregulation of related genes, leading to vigorous cell proliferation [50]. Cell adhesion molecules were also reported to be upregulated to facilitate cancer cells to migrate toward surrounding normal tissues and promote tumor invasion [51]. In some types of cancer, an oncogenic change can induce an inflammatory microenvironment that promotes the development of tumors, and “smoldering” inflammation in the tumor microenvironment has many tumor-promoting effects, such as aiding in the proliferation and survival of malignant cells, promoting angiogenesis and facilitating metastasis [52]. Pathological angiogenesis is a hallmark of cancer and concentrated efforts in this area of research are leading to the discovery of a growing number of anti-angiogenic molecules to treat cancer [53], for example, bevacizumab [54]. As for the GO terms associated with down-regulated DEGs, disarranged cell division, especially disfunction in mitotic nuclear division, might be held accountable for cellular aneuploidy, which is consistently observed in virtually all cancers [55, 56]. Additionally, dysregulation of DNA damage repair and signaling to cell cycle checkpoints, known as the DNA damage response, is associated with a predisposition to cancer and affects responses to DNA-damaging anticancer therapy [57, 58]. The result of GO analysis implied that upstream genomic abnormalities surely promoted the process of carcinogenesis, by influencing all the key biological and molecular signaling pathways through disarrangement of tumor transcriptomics. Furthermore, LUSC tumor harbored the second amount of somatic mutational burdens among all the cancer types according to TCGA mutation data, with the ranking between skin cutaneous melanoma (SKCM) and lung adenocarcinoma (LUAD, Fig. 3B). Four genes, including TTN, TP53, MUC16 and CSMD3, mutated in even more than half of the patients (Fig. 3C), and the majority of the mutational type was missense mutation (Fig. 3D). The mutational burden of LUSC indicated that the cascade of genomic catastrophe might cause large-scale transcriptional dysregulation, leading to uncontrolled tumor cell cycles.

The significant genes identified from random walk were strongly associated with sex-hormone signaling pathways (Fig. 4). All these genes were all significantly differentiated between cancer and adjacent normal tissues, independent of different genders. ERα was significantly related to all-gender and male patients’ OS, except for female. As we mentioned before, the correlation between ER and lung cancer’s prognosis varies tremendously. The profound controversy might be due to the differences in detecting methodology, i.e., which antibody is used, heterogeneous definitions of positivity, and patient population selected for research, i.e., pathology, stage, gender, and so on [12, 13, 59]. Currently, the relationship between ER and lung cancer has been widely reported in female patients and lung adenocarcinoma according to literature review. As we know, there has been no study specifically focusing upon the relationship between ER and the prognosis of male LUSC patients.

The relationship between ER and EGFR has also been widely studied in lung adenocarcinoma. EGFR has been reported to directly phosphorylate ER at specific serine residues in 87.5% of the ER-positive lung tumors [60, 61]. In addition to MEK/ERK signaling pathway, estrogen also activates the PI3K/AKT signaling pathway to promote lung cancer cell metastasis through epithelial mesenchymal transition in lung adenocarcinoma [62]. A combination of ER antagonist and EGFR tyrosine kinase inhibitor has been shown to be effective in decreasing cell proliferation and tumor growth in lung adenocarcinoma [63, 64]. These studies indicated that the intimate interaction between ER and EGFR pathways supported a rationale to use the combined therapy lung adenocarcinoma, especially in EGFR mutated patients. In our study, the interaction between ER and EGFR was also spotted to be greatly essential in LUSC, implying the combined therapy of EFGR and ER, for instance, the combination usage of cetuximab and fulvestrant, might be effective in treating LUSC patients. The correlation between ERα and OS seemed to be much stronger in male LUSC patients, and our data also confirmed this relationship through immunohistochemistry assay. More samples might be needed to verify the significant association between ERα and DFS.

## Conclusions

ERα was significantly related to a poor prognosis in LUSC, especially for male patients after radical surgery, confirmed by our immunohistochemistry data.

## Supplementary Information

**Additional file 1: Supplementary Figure S1.** Boxplot of these 12 significant genes in paired male samples. **Supplementary Figure S2.** Boxplot of these 12 significant genes in paired female samples. **Supplementary Figure S3.** OS analysis of these 12 significant genes in all-gender patients. **Supplementary Figure S4.** OS analysis of these 12 significant genes in female patients. **Supplementary Figure S5.** DFS analysis of ERα and AR in male LUSC patients. **Supplementary Figure S6.** OS analysis of ER and AR in both male and female LUSC patients, respectively using 4 independent cohorts.

## Data Availability

The datasets used and/or analysed during the current study are available from the corresponding author on reasonable request.

## Abbreviations

LUSC: Lung squamous carcinoma
NSCLC: Non-small-cell lung cancer
ER: Estrogen receptor
CNV: Copy number variation
DEGs: Differentially expressed genes
TCGA: The Cancer Genome Atlas
TSS: Transcription start site
HPRD: Human Protein Reference Database
KEGG: Kyoto Encyclopedia of Genes and Genomes
FFPE: Formalin-fixed paraffin-embedded
GO: Gene Ontology
SGOL: Segment Gain or Loss
TMB: Tumor mutational burden
BCC: Biggest connected component
AR: Androgen receptor
AdjCTX: Adjuvant chemotherapy
AdjRAD: Adjuvant radiotherapy
