# Supplementary material for: ER expression associates with poor prognosis in male lung squamous carcinoma after radical resection
Source: BMC Cancer. 2021 Sep 21;21:1043. doi: 10.1186/s12885-021-08777-6 (PMC8456567; doi:10.1186/s12885-021-08777-6)
Supplement: Supplementary file 1 — Additional file 1: Supplementary Figure S1. Boxplot of these 12 significant genes in paired male samples. Supplementary Figure S2. Boxplot of these 12 significant genes in paired female samples. Supplementary Figure S3. OS analysis of these 12 significant genes in all-gender patients. Supplementary Figure S4. OS analysis of these 12 significant genes in female patients. Supplementary Figure S5. DFS analysis of ERα and AR in male LUSC patients. Supplementary Figure S6. OS analysis of ER and AR in both male and female LUSC patients, respectively using 4 independent cohorts. [file 12885_2021_8777_MOESM1_ESM.docx]

**Supplementary files**

**
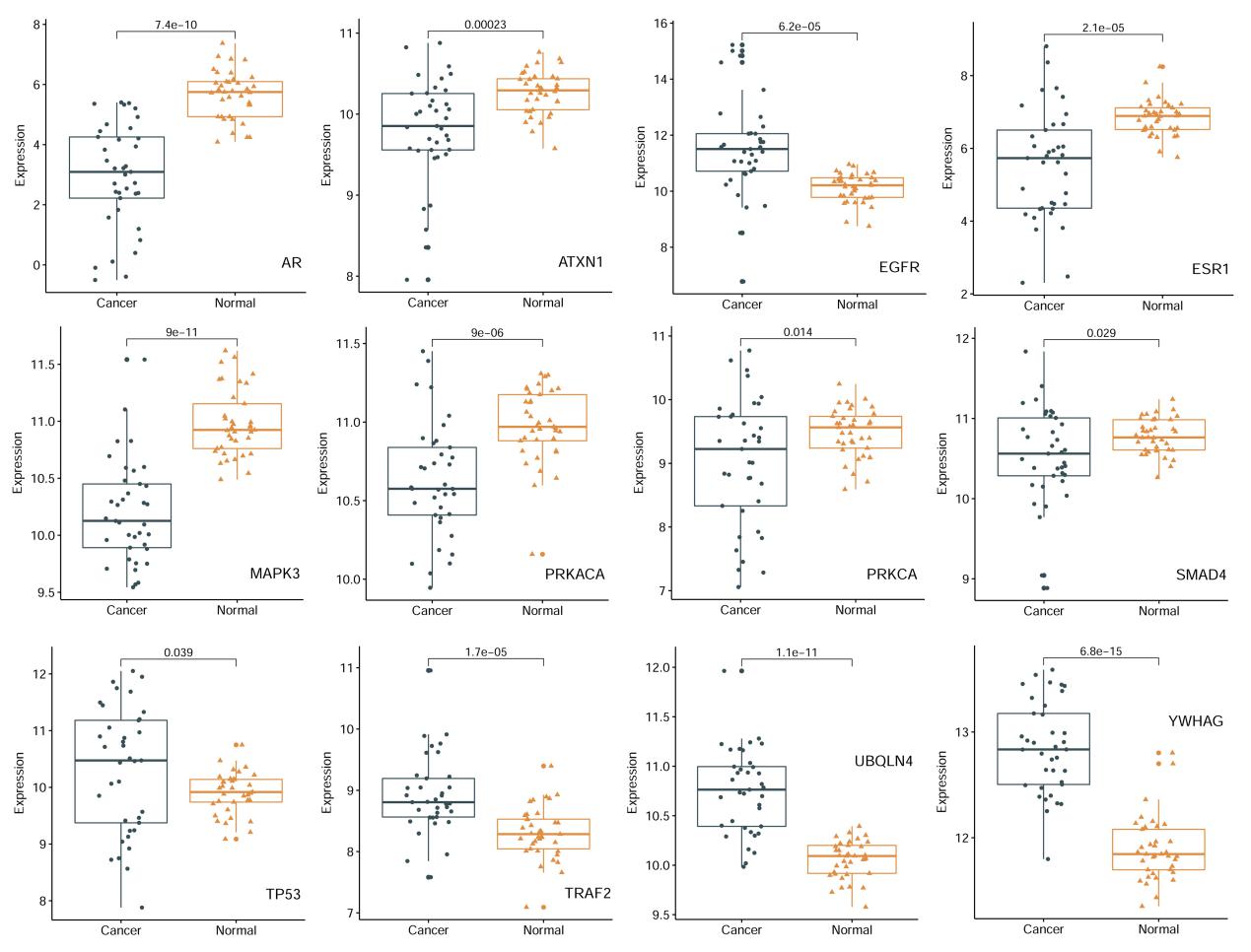
**

**Supplementary Figure S1.** Boxplot of these 12 significant genes in paired male samples.


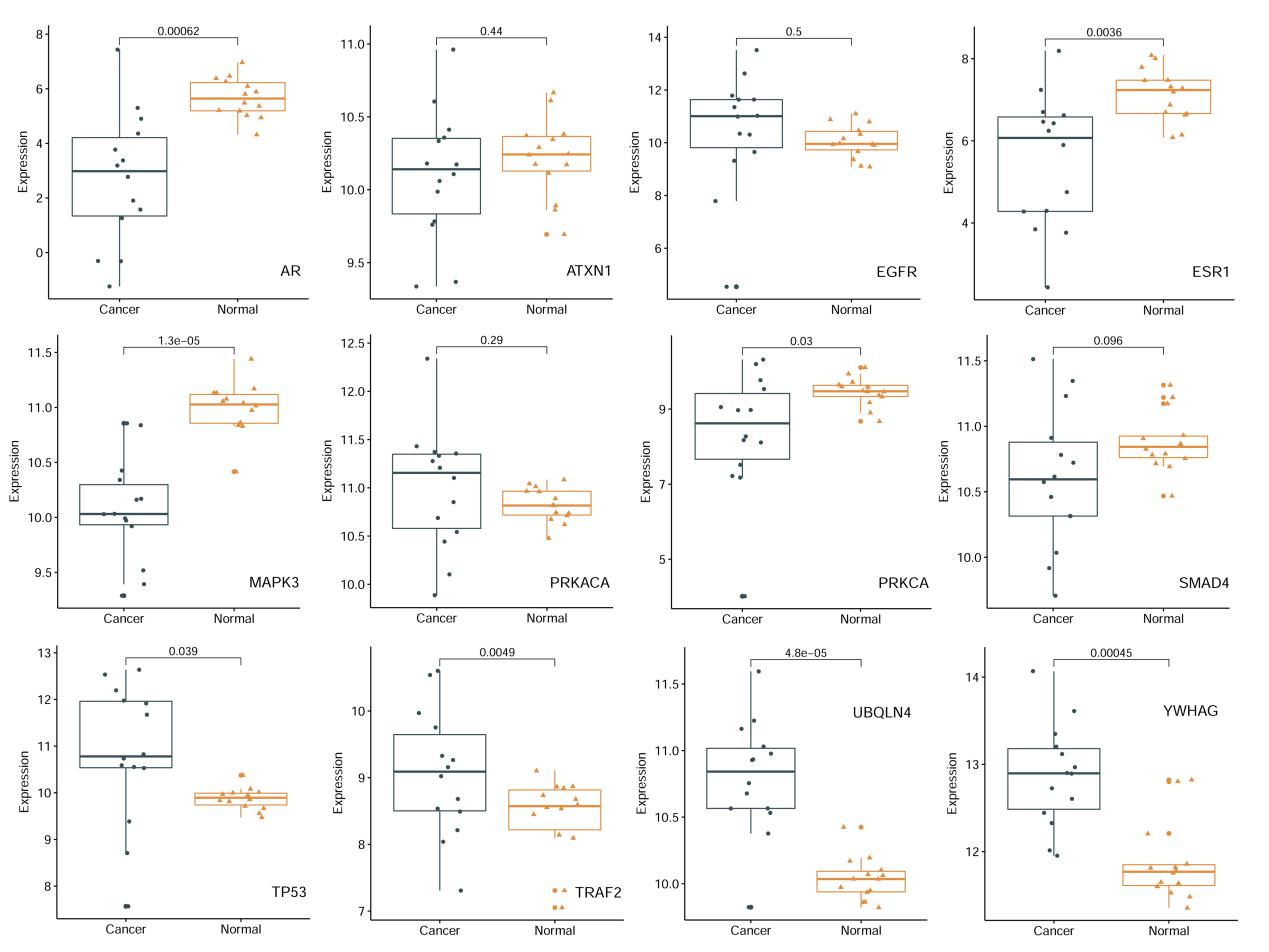


**Supplementary Figure S2.** Boxplot of these 12 significant genes in paired female samples


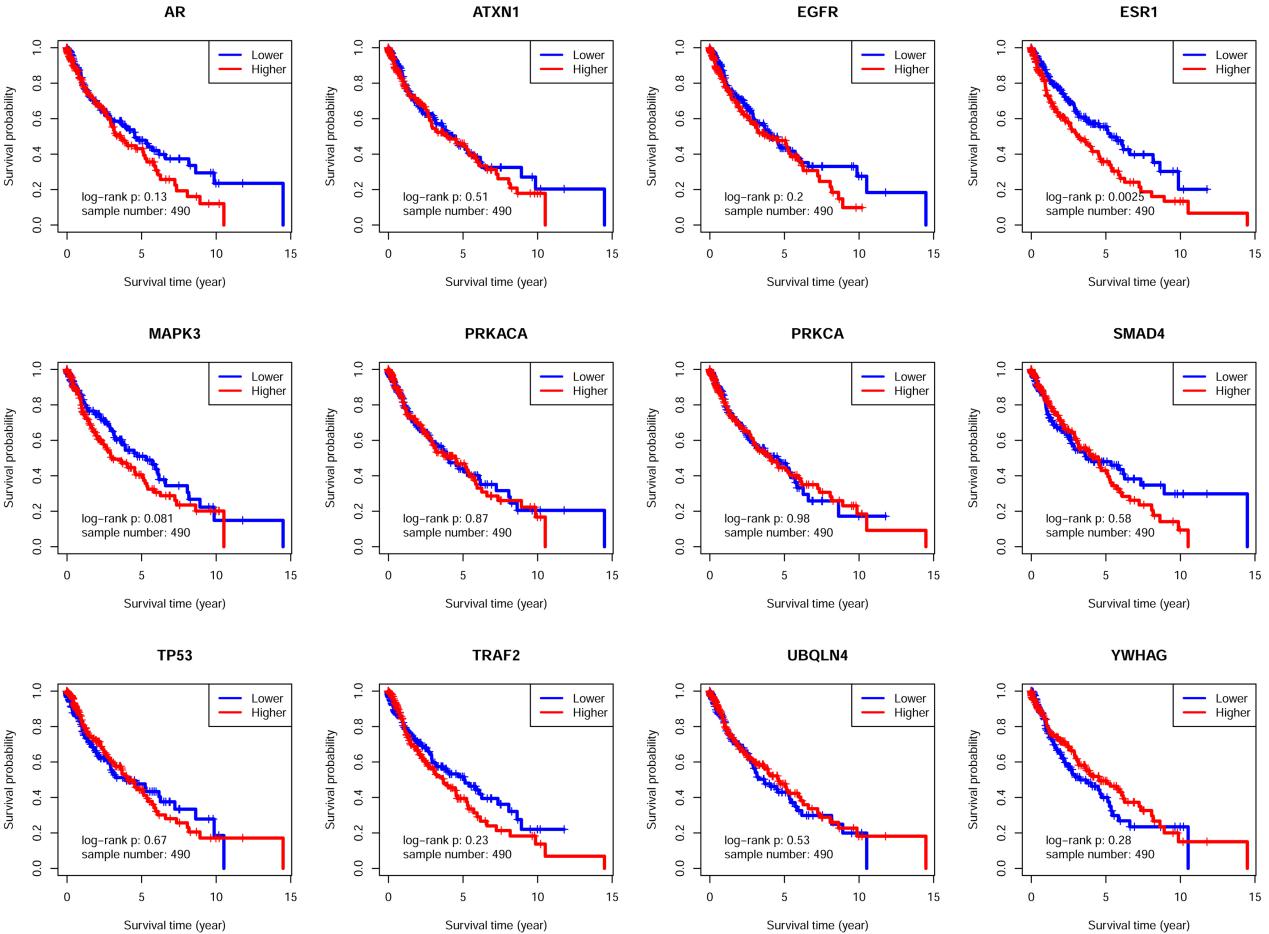


**Supplementary Figure S3.** OS analysis of these 12 significant genes in all-gender patients.


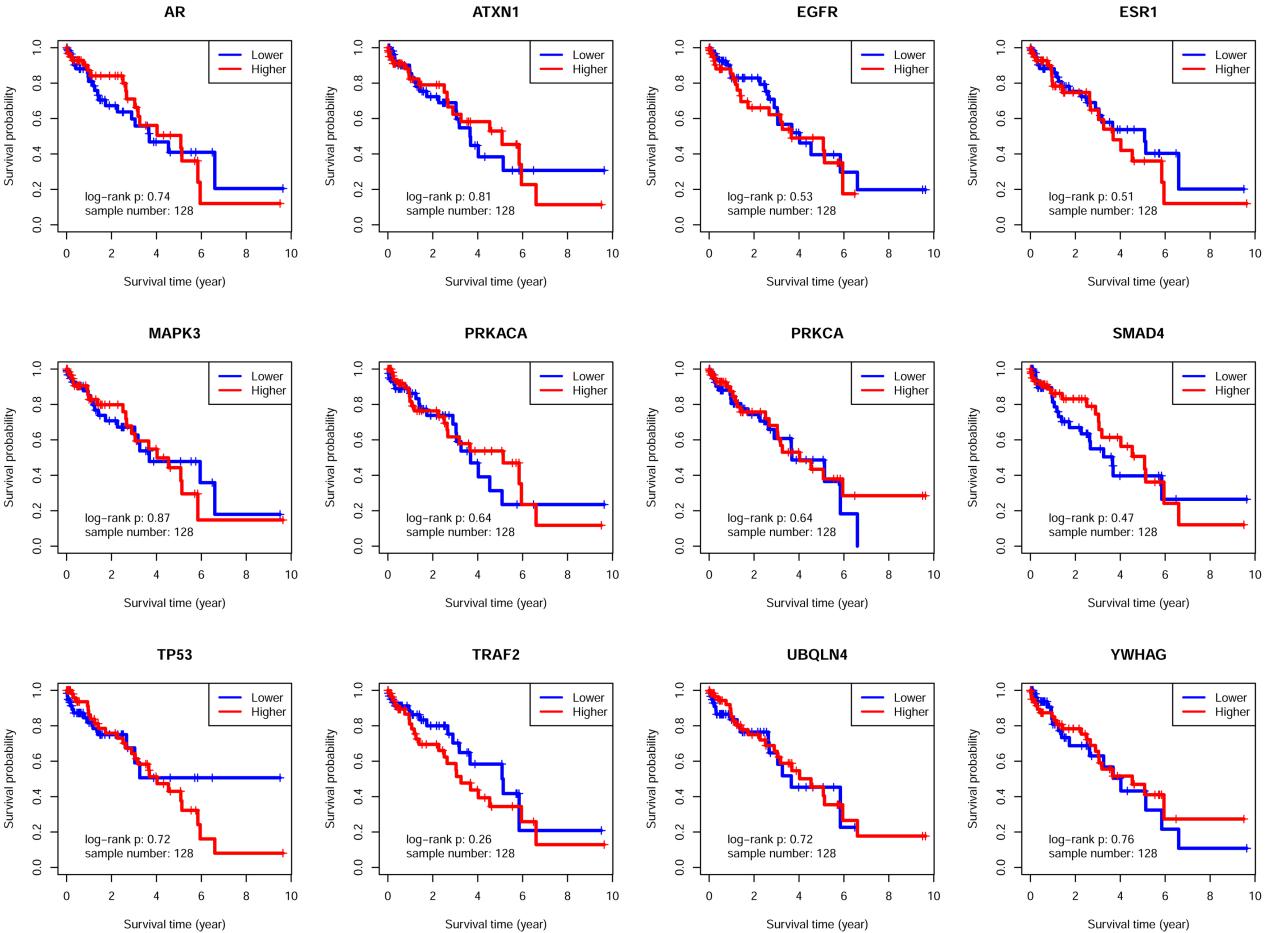


**Supplementary Figure S4.** OS analysis of these 12 significant genes in female patients.


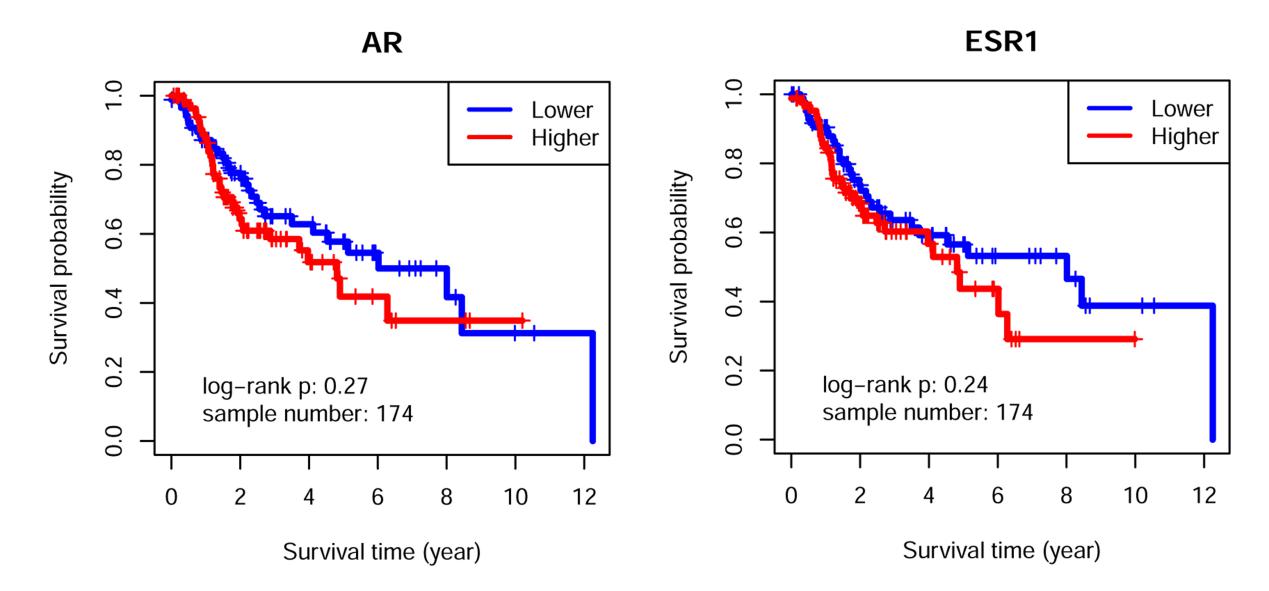


**Supplementary Figure S5.** DFS analysis of ERα and AR in male LUSC patients.


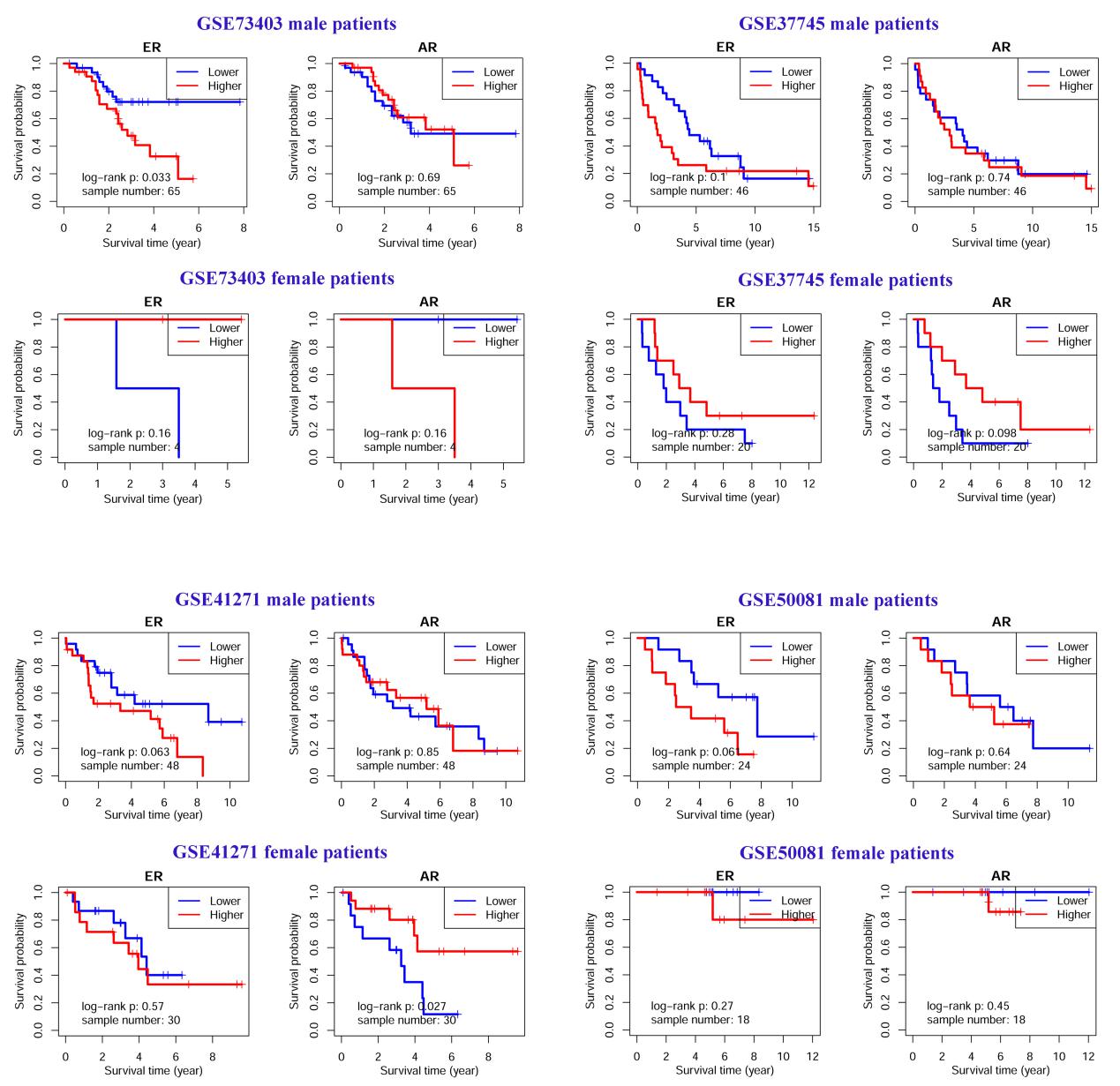


**Supplementary Figure S6.** OS analysis of ER and AR in both male and female LUSC patients, respectively using 4 independent cohorts.
